# Pedigree Analysis of Warmblood Horses Participating in Competitions for Young Horses

**DOI:** 10.3389/fgene.2021.658403

**Published:** 2021-04-15

**Authors:** Tomasz Próchniak, Kornel Kasperek, Sebastian Knaga, Iwona Rozempolska-Rucińska, Justyna Batkowska, Kamil Drabik, Grzegorz Ziȩba

**Affiliations:** Institute of Biological Bases of Animal Production, University of Life Sciences in Lublin, Lublin, Poland

**Keywords:** sport horses, inbreeding, animal breeding, pedigree, genetic diversity

## Abstract

The aim of the study was to characterize the population structure and assess the genetic diversity of warmblood horses used in the show jumping discipline. Pedigree data of 1,048 horses participating in the Polish Championships for Young Horses were analyzed. The pedigree of these animals included 12 863 individuals. The study consisted in analysis of the pedigree structure of the horses and characterization of the homozygosity and genetic diversity in the population. It was found that pedigree completeness and depth were sufficient for reliable assessment of the genetic diversity in the analyzed population. Although the average inbreeding coefficient exhibited at an acceptable level (approx. 1.01%), the increasing percentage of inbred animals seems disturbing. The results have shown that modern sport horses are derived from a small number of high-quality sires whose offspring were intensively used for breeding—bottleneck effect. In consequence, a greater part of the genetic variation reduction was observed in the non-founder generations. Given the changes in the studied population, the level of inbreeding in modern sport horses should be monitored, and pedigree data should be effectively used in selection for mating.

## Introduction

Breeding healthy high-performance sport horses that will be suitable for various horse riding disciplines and international competitions is the major goal of modern horse breeder associations. In terms of the number of events and competing horses, show jumping is the most popular discipline in Poland and worldwide^1^. The development of this discipline has stimulated the growing demand for agile horses showing an ability to cope with the increasingly difficult tasks in the parkour. There are many literature reports on the assessment of the performance and breeding value of show jumping horses of the most popular breeds in Poland and elsewhere (Rovere et al., 2016; Schubertová et al., 2016; Viklund and Eriksson, 2018). The sources of the variability of selected traits have also been well described (Ducro et al., 2009; Próchniak et al., 2015; Novotná et al., 2016; Bartolomé et al., 2018). The researchers presented the contribution of genetic (additive) and environmental variance (e.g., the influence of the rider, year and place of evaluation) to the overall variability of performance traits in horses intended for various equestrian sports. They analyzed data on the performance value of animals derived both from Training Centers (quality of individual gaits, free jumps, assessments of test riders) and sport competitions (a horse’s place in a competition, number of faults made at obstacles, time of round). A desirable addition to the current research seems to be an analysis of the dynamically changing genetic structure of the jumping horse population.

Breeding programs are mainly focused on the improvement of riding traits. This is associated with the intensive selection, which usually leads to a reduction in genetic variability and a gradual increase in inbreeding as a consequence. The increase in inbreeding should be monitored to maintain the genetic variability at an acceptable level and prevent inbreeding depression (Sevinga et al., 2004; Gómez et al., 2009; Kjöllerström et al., 2015; Todd et al., 2018). There is extensive literature discussing the level of inbreeding in various populations of riding horses and those kept as a genetic resource (Van Eldik et al., 2006; Wolc and Balińska, 2010; Binns et al., 2012; Krupa et al., 2015; Wallmann and Distl, 2017; Bussiman et al., 2018; Cecchi et al., 2018; Todd et al., 2018; Giontella et al., 2019). Borowska and Szwaczkowski (2015) reported the results of their research on the population structure and evaluation of the genetic diversity of horses participating in stationary performance tests after 100 days (sires) and 60 days (mares) of training in training centers. However, there are no similar studies on animals assessed during the Polish Championships for Young Horses—a popular method for assessment of the performance value of sport horses in Poland. During the Championships the most popular domestic breed is the Polish Halfbred Horse. This population of horses, which is characterized by diverse genotypes and phenotypes (Lewczuk, 2005), often derive from mating Polish mares with highly genetic valued foreign sires (import of genetics). For last twenty years, there has been an intensive increase in the participation of foreign sport breed horses both in competitions and reproduction as well. As a consequence, there was a decrease in the number of horses of domestic breeds: Malopolska and Wielkopolska (Pietrzak and Próchniak, 2014; Próchniak et al., 2014), which may lead to a decrease in genetic diversity. On the other hand maintenance of high genetic variation in a population is essential for the achievement of breeding progress within the improved traits. The aim of the study was to characterize the population structure and assess the genetic diversity of warmblood horses evaluated in the Polish Championships for Young Horses in Show Jumping.

## Materials and Methods

Pedigree data of 1,048 horses (724 sires and 324 mares) taking part in the show jumping competition in the Polish Championships for Young Horses in 2006–2015 (reference population) were analyzed (Table 1). Animals from the reference population were born in 2009–2011.

**TABLE 1:** Reference population size with the birth year, studbook record, origin, and sex.

**Birth year**	**Studbook^a^**								**Origin^b^**		**Gender**		**Σ**
	**sp**	**wlkp**	**m**	**han**	**hol**	**old**	**kwpn**	**other**	**P**	**F**	**♂♂**	**♀♀**	
1999	11	1	2	3	0	0	0	0	16	1	10	7	17
2000	20	2	3	13	3	2	0	1	32	12	30	14	44
2001	58	2	1	23	5	0	2	3	65	29	73	21	94
2002	46	7	1	41	5	1	3	2	67	39	75	31	106
2003	48	9	2	44	3	2	2	0	78	32	79	31	110
2004	52	8	3	43	1	1	1	1	73	37	74	36	110
2005	36	7	1	37	3	2	1	0	50	37	63	24	87
2006	40	8	2	51	1	1	0	3	53	53	77	29	106
2007	44	7	1	54	4	0	0	0	56	54	73	37	110
2008	30	2	0	36	1	0	0	4	38	35	52	21	73
2009	28	5	2	29	6	4	2	3	40	39	51	28	79
2010	37	6	1	1	10	2	7	8	45	27	40	32	72
2011	17	5	1	0	9	3	2	3	23	17	27	13	40
Σ	467	69	20	375	51	18	20	28	636	412	724	324	1,048

*^*a*^sp, Polish Halfbred Horse; wlkp, Wielkopolska breed; m, Malopolska breed; han, Hanoverian breed; hol, Holsteiner breed; old, Oldenburg breed; kwpn, Royal Dutch Sport Horse; other, horses of other breeds.*

*^*b*^P, Polish breeding; F, foreign breeding.*

**FIGURE 1:**
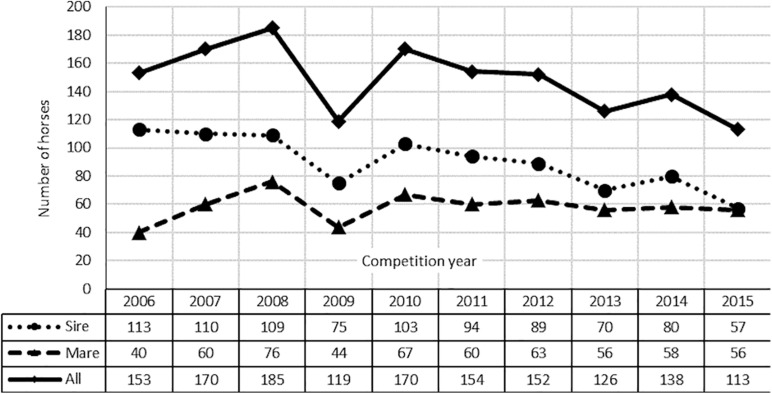
Number of horses starts in Polish Championships for Young Horses in 2006–2015.

The pedigrees of these horses contained 12 863 individuals (3,469 sires and 8,383 mares) and reached a maximum of 15 generations (on average 6). The number of population founders, i.e., individuals without known parents, and the number of non-founders, i.e., animals with at least one known parent. Even though belonging to the different studbooks, the examined horses were analyzed as one population. Such an approach is supported by the existence of common ancestors of modern sports horses and the constant exchange of horses between different studbooks. Moreover, all the horses underwent the same utility value assessment (Polish Championship of Young Horses). The number of starts of the analyzed horses (1,480) is shown in Figure 1.

The population was characterized using the measurements used also by Borowska and Szwaczkowski (2015; Table 2). The following characteristics were estimated: the percentage of animals with both known parents (as a proportion), discrete generation equivalents—*g*_*e*_ (Sölkner et al., 1998), the pedigree completeness—*C*_*p*_ (Cassell et al., 2003), the generation interval—*L* (James, 1977; Hill, 1979), individual inbreeding coefficients—*F*_*i*_ (Colleau, 2002; Gómez et al., 2009), individual increase in inbreeding—*ΔF_*i*_* (Colleau, 2002; Cervantes et al., 2008; Gutiérrez et al., 2008), the effective population size—$\bar{N_{e}}$, the number of individuals in an ideal population whose offspring constitute the next generation (Colleau, 2002; Cervantes et al., 2008; Gutiérrez et al., 2008), the founder equivalent—*f*_*e*_, that denote the numbers of equally contributing founders that would result to the same level of genetic diversity in the current population (Lacy, 1989; Sargolzaei and Colleau, 2006), the founder genome equivalent—*f*_*ge*_, indicating how many founders would be required to produce the same genetic diversity that found in the population if all founders contributing equally and no founder alleles were lost by drift under random mating (Lacy, 1989; Sargolzaei and Colleau, 2006), non-founder equivalent—*N*_*enf*_, that considers only the effect of genetic drift in non-founder generations (Caballero and Toro, 2000), the genetic diversity—*GD* (Lacy, 1989, 1995) and *GD*^∗^ (Caballero and Toro, 2000), taking into account the bottleneck effect and genetic drift and including only founder equivalent, respectively, the difference between *GD*^∗^ and *GD* (Caballero and Toro, 2000). The contribution of founder alleles in the mean inbreeding coefficient and coancestry was calculated using three component vectors (Sargolzaei and Colleau, 2006): vector *v* containing contributions of founders’ genes, vector *m* containing Mendelian sampling variances of ancestors, vector *u* containing contributions of genes of nodal common ancestors. The results also present the contribution of founders in mean population inbreeding (*vf*) calculated according to the formula: $v\,f=\frac{F\%}{\sum v_{1}}\times 100$.

**TABLE 2:** The statistical measurements used for the population characterization.

**Parameter**	**Formula**	**Description**
Discrete generation equivalents	${\text{g}}_{\text{e}}=\frac{1}{\text{N}}\,\sum_{\text{j}=1}^{\text{N}}\sum_{\text{i}=1}^{\text{n}}\frac{1}{2^{{\text{g}}_{\text{ij}}}}$	*n*_*j*_—number of known ancestors of the *j*-the individual *g*_*ij*_—the number of generations between the *i*-the ancestor and the *j*-the animal *N*—number of animals in the reference population
Pedigree completeness	$c_{p}=\frac{a_{k}}{\sum_{i=1}^{g}2^{i}}$	*a*_*k*_—number of known individuals in five generations of ancestors.
The generation interval	$L=\frac{L_{s\,s}+L_{s\,m}+L_{m\,s}+L_{m\,m}}{4}$	*L*—the average age of individuals at offspring birth, *s*, sire; *m*, mare Performed for four paths: father, father-daughter, mother-son, and mother-daughter
Individual increase in inbreeding	△F_i_=1−1−F_i_g*_e_*−1	*F*_*i*_—individual inbreeding coefficient *g*_*e*_—discrete generation equivalent
Effective population size	$\bar{{\text{N}}_{\text{e}}}=\frac{1}{2\,\bar{△\,{\text{F}}_{i}}}$	Calculated based on calculated from $\bar{△\,F}$, which is an arithmetic mean *△F* of analyzed individuals, *ΔF-* individual increase in inbreeding
Founder equivalent	${\text{f}}_{\text{e}}=\frac{1}{\sum \left({\text{p}}_{\text{i}}^{2}\right)}$	*p*_*i*_- proportion of genes of the *i*-th founder in the reference population
Founder genome equivalent	$f_{g\,e}=\frac{1}{\sum \left(\frac{p_{i}^{2}}{r_{i}}\right)}$	*p*_*i*_-proportion of genes of the *i*-th founder in the reference population *r*_*i*_- expected proportion of genes of the *i*-th founder that are preserved in the reference population
Non-founder equivalent	${\text{N}}_{\text{enf}}={\left(\frac{1}{{\text{f}}_{\text{ge}}}-\frac{1}{\text{fe}}\right)}^{-1}$	*f_*ge*_*—founder genome equivalent*f_*e*_*—founder equivalent
Genetic diversity (it takes into account the bottleneck effect and genetic drift)	$\text{GD}=1-\frac{1}{2\,f_{\text{ge}}}$	*f_*ge*_*—founder genome equivalent
Genetic diversity (only includes founder equivalent)	${\text{GD}}^{*}=1-\frac{1}{2\,f_{\text{e}}}$	*f_*g*_*—founder equivalent
The difference between GD* and GD	${\text{GD}}^{*}-\text{GD}=1-\frac{1}{2\,N_{\text{enf}}}$	*N*_*enf*_ was calculated as above

The parameters were estimated with software CFC 1.0 (Sargolzaei and Colleau, 2006), Endog 4.8 (Gutiérrez and Goyache, 2005), and EVA (Berg et al., 2006).

## Results

### Pedigree Structure

According to the studbook (pedigree data), the greatest number of horses taking part in the show jumping competition in the Polish Championships for Young Horses in 2006–2015 represented the Polish Halfbred Horse breed. The animals of this breed are selected for performance traits that are relevant in sports, e.g., show jumping. Although they are not homogeneous in terms of origin and often derive from Polish mares mating with highly valued foreign sires, they are entered into a separate studbook, as is the case with other Polish horse breeds. There were relatively small numbers of native halfbred Malopolska and Wielkopolskahorses. Although these animals have been bred as general-purpose horses for many years, the current breeding programs are focused on selection for sport performance traits. The Malopolska breed was based on native horses that for centuries have been mated with purebred sires and halfbred European sires lines such as Schagya, Gidran, Dahoman, Amurath, Gazlan, Furioso, Przedswit, and Nonius. The Wielkopolska breed was developed via refinement of local herds with East Prussian and Trakehner horses and, to a lesser extent, with domestic and foreign half-bred animals. Thoroughbred horses have contributed greatly to the creation and improvement of the breed. The foreign animals represented mainly the most popular equestrian breeds, e.g., Hanoverian, Holsteiner, Oldenburg, and Royal Dutch Warmblood Horses (Table 1).

**FIGURE 2:**
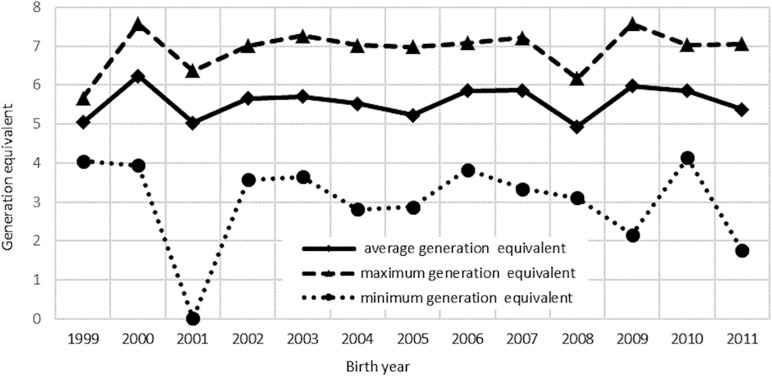
Average, minimum, and maximum of discrete generation equivalents of the studied population.

Details of the pedigree structure are presented in Table 3 and Supplementary Table 1. The analysis involved 12 863 pedigrees of horses, including 1,621 founders. There were 11,242 non-founders, which accounted for 87% of all animals in this group (Table 3). Both parents of all horses from the reference population were known. The longest ancestral path *(LAP)* was 15 generations (Supplementary Table 1), while the average number of ancestor generations of the total population was 4.25. In total, 1,496 pedigree lines and 365 full-sib groups were detected. The average number of discrete generation equivalents in the reference population was 5.65, with a maximum of 7.26 in the group of horses born in 2000 (Figure 2). The average pedigree completeness coefficient for 5 generations of ancestors of individuals from the reference population was 82.83%. Table 4 shows the generation interval based on the pedigree of the total population. It was on average 9.99 years. The shortest value was found for the mother-son relationship (8.95 years) whereas the father-son relationship had the longest interval (11.08 years).

**TABLE 3:** Pedigree structure in the analyzed population.

**Parameter**	**Sex**		**Σ**
	**♂♂**	**♀♀**	
Reference population	724	324	1,048
Number of individuals in the pedigree	4,162	8,701	12,863
Number of individuals with offspring	3,469	8,383	11,852
Number of individuals without offspring	693	318	1,011
Number of founders	476	1,145	1,621
Number of non-founders	3,686	7,556	11,242
Number of non-founders with both known parents	2,848	6,820	9,668

**TABLE 4:** Generation interval in the analyzed population, standard deviation (SD), and error (SE).

**Relationship**	***N***	**Interval**	***SD***	***SE***
Father—son	3,614	11.081	5.601	0.104
Father—daughter	7,360	10.617	5.301	0.063
Mother—son	3,432	8.949	4.138	0.079
Mother—daughter	6,504	9.207	4.422	0.056
Total	20,910	9.985	4.994	0.036

### Homozygosity in the Population

The percentage of inbred animals in the total population (all individuals in the pedigree) was estimated at 28.5% individuals, and the level of inbreeding coefficient was in the range of 0–31.35%, with the inbreeding coefficient greater than 20% in only 28 animals. In the reference population the value of the inbreeding coefficient, in 1999–2011 years, was higher than zero in as many as 78% of sires and 81% of mares (Supplementary Table 2). Although the level of inbreeding coefficient in the reference population did not exceed 25%, the percentage of inbred animals increased in consecutive years and exceeded 90% animals in 2009–2011 (Supplementary Table 2 and Table 5). In the reference population the average inbreeding coefficient in the analyzed period increased and ranged from 0.35 to 1.41% (on average 1.01%), with a maximum value recorded in 2009 (Table 5).

**TABLE 5:** Effective number of founders (f_*e*_), founder genome equivalent (f_*ge*_), effective number of non-founders (N_*enf*_), average inbreeding coefficients (F%), average coancestry ($\bar{\text{f}}\%$), average individual increase in inbreeding (ΔF_1_%), and effective population size ($\bar{{\text{N}}_{\text{e}}}$) for the groups by birh year.

**Birth year**	**f_*e*_**	**f_*ge*_**	**N_*enf*_**	**F%**	$\bar{\text{f}}\%$	**ΔF_*i*_%**	$\bar{{\text{N}}_{e}}$
1999	156	13	14	0.345	3.868	0.079	633
2000	159	24	28	0.595	2.069	0.123	407
2001	185	40	52	0.543	1.235	0.109	458
2002	181	43	56	0.899	1.166	0.229	219
2003	166	39	51	0.921	1.276	0.190	263
2004	183	42	54	0.970	1.201	0.200	250
2005	132	30	39	1.260	1.652	0.299	167
2006	127	31	41	0.991	1.603	0.220	227
2007	112	28	38	1.121	1.756	0.239	209
2008	116	26	34	1.007	1.901	0.217	230
2009	113	26	34	1.410	1.902	0.352	142
2010	122	28	36	1.321	1.800	0.254	197
2011	87	18	23	1.270	2.751	0.315	158
Total	152	53	81	1.007	0.943	0.220	227

**FIGURE 3:**
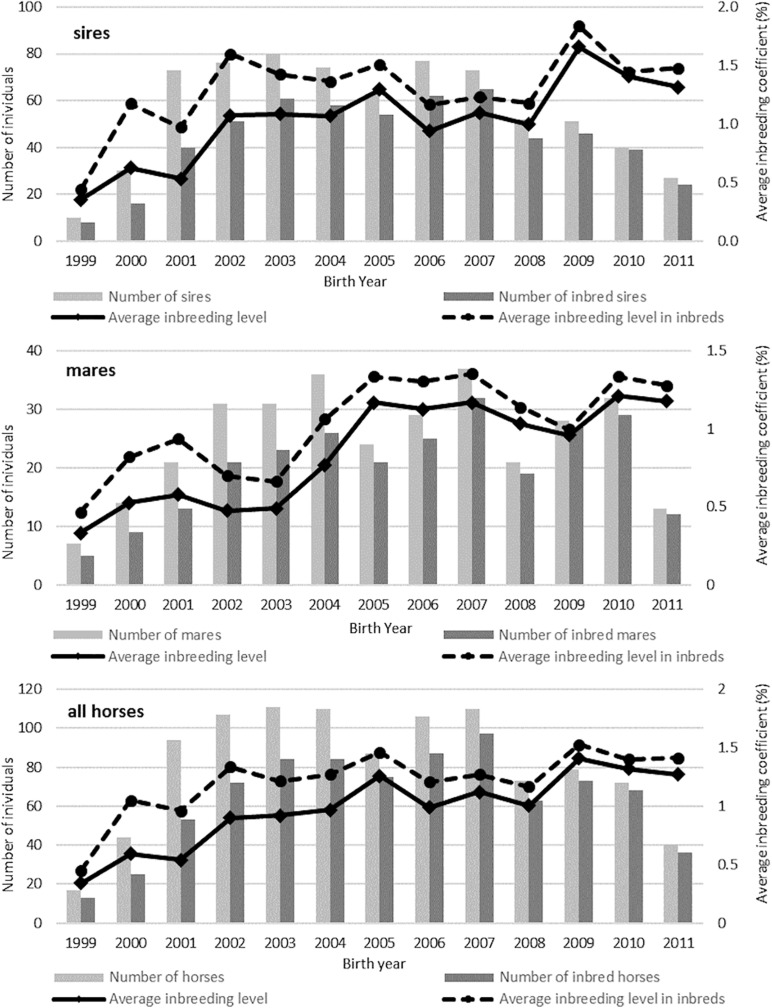
Number of inbred horses and the average inbreeding coefficients (F%) in consecutive years for sires, mares, and all horses.

The trends in changes in the inbreeding coefficientlevel regarding animal sex and numbers in subsequent years are shown in Figure 3. It was observed that the fluctuations in the inbreeding coefficientlevel were not always associated with the number of animals from the same birth year. Table 6 shows the average inbreeding coefficient in the breed groups. The highest level of inbreeding coefficientwas observed in the German horses, i.e., the Holsteiner (2.97%) and Hanoverian (1.26%) breeds. The lowest inbreeding coefficientlevel was found in the Polish breeds, i.e., Malopolska (0.34%), Wielkopolska (0.56%), and Polish Halfbred horses (0.66).

**TABLE 6:** Effective number of founders (f_*e*_), founder genome equivalent (f_*ge*_), effective number of non-founders (N_*enf*_), average inbreeding coefficients (F%), average coancestry ($\bar{\text{f}}\%$),average individual increase in inbreeding (ΔF_*i*_%), and effective population size ($\bar{{\text{N}}_{\text{e}}}$) for the groups by studbook.

**Studbook^*a*^**	**f_*e*_**	**f_*ge*_**	**N_*enf*_**	**F%**	$\bar{\text{f}}\%$	**ΔF_*i*_%**	$\bar{{\text{N}}_{e}}$
sp	188	53	74	0.655	0.945	0.100	500
wlkp	175	35	44	0.559	1.427	0.092	543
m	103	11	12	0.343	4.516	0.057	883
han	114	38	56	1.259	1.325	0.200	250
hol	37	10	15	2.970	4.797	0.458	109
old	80	10	12	1.209	4.815	0.212	236
kwpn	96	13	15	0.760	3.968	0.141	354
other	163	20	22	1.005	2.560	0.154	325

*^*a*^sp, Polish Halfbred Horse; wlkp, Wielkopolska breed; m, Malopolska breed; han, Hanoverian breed; hol, Holsteiner breed; old, Oldenburg breed; kwpn, Royal Dutch Sport Horse; other, horses of other breeds.*

**FIGURE 4:**
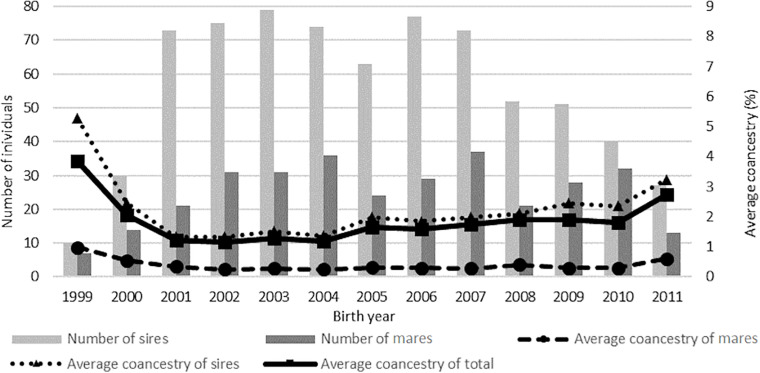
Average coancestry of the studied populations.

The average coancestry value in the total population was 1.86%, with a higher percentage in the group of mares (3.54%) than in the group of sires (2.26%). The highest average coancestry value in the analyzed population was noted in the group of horses born in 1999. It was also found that the average relatedness in the birth years was inversely proportional to the number of horses (Figure 4). The average coancestry value in the subsequent years and breeds is presented in Tables 5, 6.

The highest average relatedness was found in the group of German horses registered in the Holsteiner (9.60%) and Hanoverian (9.63%) studbook and in the Malopolska breed (9.03%) (Table 7). This indicator had the lowest value in the group of the Polish Halfbred animals (1.89%). The highest relatedness level was recorded between the Holsteiner and Oldenburg horses (4.93%) as well as the Holsteiner and Dutch breeds (3.36%) (Table 7). The lowest average relatedness level was noted between the Malopolska and foreign breeds (0.02–0.17%). The average relatedness between the horses registered in the Polish studbooks was in the range of 0.26–1.09%, with the highest value noted between the sp horses and Wielkopolska breed (1.09%) (Table 7).

**TABLE 7:** Average numerator relationships (%) within breed groups (diagonal) and between breed groups (above diagonal).

**Studbook^a^**	**sp**	**wlkp**	**m**	**han**	**hol**	**old**	**kwpn**	**other**
sp	1.890	1.091	0.263	1.617	2.416	1.886	1.553	1.199
wlkp		2.854	0.395	1.189	1.920	1.682	0.978	1.062
m			9.032	0.088	0.017	0.041	0.016	0.171
han				2.649	3.955	2.922	2.211	1.800
hol					9.594	4.933	3.361	3.002
old						9.630	2.212	2.361
kwpn							7.935	1.619
other								5.120

*^*a*^sp, Polish Halfbred Horse; wlkp, Wielkopolska breed; m, Malopolska breed; han, Hanoverian breed; hol, Holsteiner breed; old, Oldenburg breed; kwpn, Royal Dutch Sport Horse; other, horses of other breed.*

On average, inbreeding (*ΔF_*i*_*) increased by 0.220% per year. The increasing trend of this phenomenon was observed, with a maximum value of 0.315% in the last year of the study. The Ne value in the analyzed period ranged from 142 to 633 individuals. There successive decrease of the effective population size was inversely proportional to the inbreeding coefficientlevel in the reference population.

### Genetic Diversity in the Population

It was found that the 10 founders presented in Supplementary Table 3, explained *vf* = 55% of the inbred in the reference population. The first three founders: Ladykiller, Rantzau, and Ramzes contributed to *vf* = 13, 11, and 7%, respectively, of the actual inbred of the horses.

The parameters of genetic diversity obtained based on the probability of origin of alleles in the subsequent birth years of the reference population are shown in Table 5. Generally, the effective number of founders and the effective number of founder genomes varied over the period studied and declined with the decrease in the number of animals in a given year. The highest value of these parameters was noted in 2001–2004, i.e., a period with the highest number of horses. The effective number of founders was 152 and the effective number of founder genomes was 53 in the entire reference population.

The analysis of the breed groups showed the highest effective number of founders (188) in the group of Polish Halfbred horses and the lowest value (37) in the Holsteiner horses. Despite the low frequency, the effective number of founders in the Malopolska horses was 103.

**FIGURE 5:**
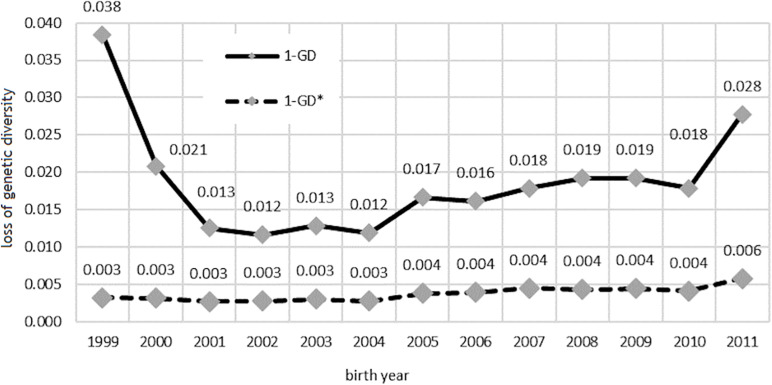
Loss of genetic diversity due to unequal founder contribution, bottlenecks, and genetic drift (1-GD) and only due to unequal founder contribution (1-GD*) in the studied population.

Changes in the level of genetic diversity are shown in Figure 5. The loss of genetic variation caused exclusively by the unequal number of founders in the population *(1-GD^∗^)* almost doubled in the analyzed period and reached 0.6% in 2011.

The loss of genetic variation caused by the uneven proportion of founders, genetic drift, and bottlenecks effect had higher values and was especially high in the birth year group represented by a small number of horses.

## Discussion

Precise and complete pedigrees closely associated with the accuracy of estimation of genetic parameters as well as the assessment of the breeding value are an important part of breeding work (Cassell et al., 2003). It was assumed that the average number of discrete generation equivalents *(g_*e*_)* of 5.65 in the reference population (Figure 2) indicates satisfactory pedigree completeness and allows estimation of homozygosity (Curik et al., 2003). This value was almost identical (*g*_*e*_ = 5.7) to that reported by Cervantes et al. (2008), who used the Cassel method for determination of pedigree completeness in Arabian horses from Spain. It was higher than the *g*_*e*_ value proposed by Wolc and Balińska (2010), who analyzed inbreeding coefficientin the Polish primitive breed (*g*_*e*_ = 4.7). The pedigree completeness coefficient of 82.83%, calculated using the Cassel method (Cassell et al., 2003), is similar to values of the parameter reported by other researchers (Zechner et al., 2002; Siderits et al., 2013).

The generation interval of 9.9 is similar to that estimated in Icelandic horses: 9.7 (Hugason et al., 1985), Trakehner horses: 10.2 (Teegen et al., 2009), and Friesian horses: 9.6 (Sevinga et al., 2004). Similarly, the generation interval estimated in various horse populations in Slovakia ranged from 9.96 to 12.27 (Pjontek et al., 2012). Noteworthy, the largest generation interval between fathers and sons does not coincide with the data reported by Hugason et al. (1985), who showed that it was the shortest interval in this relationship. Hugason et al. (1985) analyzed a population of Icelandic toelter horses, which are not evaluated for their sporting value and consequently start reproducing early. In the case of certified sires, reproduction often starts only after the end of their sporting career.

The coefficient of coancestry and the average numerator relationships were used for assessment of the degree of relatedness in the year-of-birth and breed groups. The values of the coancestry and inbreeding coefficients in the studied population were significantly higher than in the study conducted by Borowska and Szwaczkowski (2015) in horses assessed during performance tests in Training Centers. Similarly, the higher relatedness in the group of mares (3.54%) than in the group of sires (2.25%) demonstrated in the present study is not in agreement with the results reported by these authors. This may be due to the larger effective number of founders in the population studied by Borowska and Szwaczkowski (2015).

*ΔF_*i*_*is recommended by some researchers (González-Recio et al., 2007; Gutiérrez et al., 2008) as an alternative measure of inbreeding taking into account the depth of the individual’s pedigree. *ΔF_*i*_* has been successfully used by researchers to analyze horses’ pedigrees (Cervantes et al., 2008).

Although the inbreeding phenomenon is currently inevitable, the inbreeding coefficient in the reference population did not exceed 1.41%, with an average of only 0.46%. Within the breed groups, the highest average inbreeding coefficient was noted in the Holsteiner horses (2.97%), which are often used in the show jumping discipline at present. In comparison, a similar average inbreeding coefficient (2.9%) was recorded in a population of native Italian horses (Giontella et al., 2019) and in a group of sport horses from Brazil: 3.30% (Medeiros et al., 2014). Higher average inbreeding coefficient levels were recorded by Duru (2017) in a group of Turkish Arabian Horses (4.90%) and by Vicente et al. (2012), who estimated the value of this parameter at as much as 11.34% in Lusitana horses.

A relatively low average level of inbreeding coefficientwas found in the Malopolska group (0.34%), where the mean values of coancestry between the individuals (4.5%) were similar to those in the Oldenburg and Holsteiner horses. It can be assumed that the high coancestry coefficient in the Malopolska group horses is caused by the decrease in the population size. Importantly, this breed has never been selected strictly for sport use and the number of sires transferring a high level of sport performance traits is low. Hence, Malopolska horses participating in show jumping competitions have a large percentage of common ancestors, which may be reflected in increased inbreeding coefficientlevels in this population in the near future. It is commonly believed that inbred in horse populations is not a real problem, as it is several times lower than in other livestock animals, e.g., dairy cattle (Hofmannová et al., 2019). Nevertheless, the increase in inbreeding coefficientshown in Figure 3 seems to be disturbing, since it affects up to 90% of horses in some years. The average growth in inbreedingcoefficient, which increases during this period by approximately 25%, is equally alarming. It can be assumed that the inevitable progress in equestrian sports resulting in tightening of the selection criteria may enhance the tendency, which in turn will contribute to a partial loss of genetic variation in horse populations. It should be borne in mind that increased inbreeding leads to inbreeding depression in the long run, which has already been observed in Thoroughbred horse populations (Todd et al., 2018) and Arabian horses (Comparini et al., 2019). Investigations of the effect of inbreeding on sport performance traits in local environmental conditions seem advisable. Furthermore, it is necessary to control the level of inbreedingcoefficient; hence, it would be reasonable to include pedigree information in a selection of pairs for mating.

The greatest contribution to the reference population was found for the Thoroughbred stallions Ladykiller and Rantzau as well as Ramzes, i.e., an Anglo-Arabian halfbred stallion bred in Poland. The high contribution of these stallions to the modern population of sport horses was also confirmed in other studies of Polish (Borowska et al., 2011; Borowska and Szwaczkowski, 2015), German—Holsteiner (Roos et al., 2015), and Brazilian sport horses (Medeiros et al., 2014). The genetic contribution of the most popular sire Ladykiller to the population was 3.75%. Its offspring, especially Landgraf I, were used for reproduction in many horse breeder associations; hence, this horse is the ancestor of many modern sport horses. The Anglo-Arabian horse Ramzes is the founder of the famous German sport horse line from which e.g., Ramiro Z and Ratina Z originate. The effective population size should be considered as the size of an ideal theoretical population that would lose heterozygosity at the same rate at which the loss of heterozygosity occurs in the real studied population. $\bar{N_{e}}$is dependent on e.g., the polygamy ratio, number of offspring, fluctuations in the size of subsequent generations, and overlap of generations. The effective population size decreasing by almost 75% in the analyzed period is probably associated with the increase in inbreeding as well (Table 5). The highest effective population size was observed in the Malopolska horses (883) exhibiting the lowest average inbreeding coefficient, and the lowest value of this parameter was noted in the Holsteiner horses, which were characterized by the highest average inbreedingcoefficient.

The genetic diversity parameters presented in Table 5 characterize the expected heterozygosity in the analyzed population and are widely used even when a small number of generations in the population is known (Boichard et al., 1997). Genetic diversity parameters are specified based on the probability of origin of the allele. It was noticed that the founder equivalent (*f*_*e*_) decreased in the subsequent years, whereas the founder genome equivalent (*f*_*ge*_) and the effective number of non-founders (*N*_*enf*_) were the lowest in the years with the lowest number of horses. The lowest level of genetic diversity was observed in the group of horses with the highest inbreeding coefficient (Holsteiner and Oldenburg). Unequal contribution of founders to the genetic pool of the population was noted. The decreasing *f*_*e*_ parameter (Table 5) may be a result of the preference for horses from a narrow group of sires in equestrian sports. In turn, the effective number of non-founders *(N_*enf*_)*, which was higher than the founder genome equivalent *(f_*ge*_)*, indicated cumulative genetic drift in the non-founder generations. The loss of genetic diversity (Figure 5) was associated with the unequal founder contribution *(1-GD^∗^)*and the genetic drift and bottleneck effect *(1-GD)*. This may be associated with the small number of sires used for reproduction in a certain period, which resulted in numerous offspring used in breeding sport horses. In recent decades, the transfer of genetic information has been facilitated by the development of artificial insemination techniques. Domestic mares are often mated with the best stallions with a high breeding value. In the case of appropriate breeding work, this can increase the pool of desired genes in the population. On the other hand, there is an obvious risk of inbreeding and the bottleneck effect in the population, which may result in drastic loss of genetic variation. The results of our work complement the knowledge about the genetic variability of sport horses in Poland, also presented by Borowska and Szwaczkowski (2015). Although, as a rule, horses evaluated in Training Centers do not participate in Polish Championships for Young Horses, both groups of animals are characterized by a mutual origin and the same problems related to the loss of genetic variability.

## Conclusion

In conclusion, it was found that the pedigree completeness and depth were sufficient for a reliable assessment of the genetic diversity of the analyzed population. The average generation interval was 10 years with the longest value between fathers and sons. A greater part of the genetic variation reduction was observed in the non-founder generations, which may be a result of mating domestic mares with a small number of foreign sires with high-performance value, which in turn had numerous offspring used for reproduction - the bottleneck effect. The thoroughbred stallions Ladykiller and Rantzau and the Anglo-Arabian stallion Ramzes are the main founders of the studied population, which confirms that modern sport horses are derived from a small number of high-quality sires whose offspring were intensively used for breeding, as shown in literature. Although the value of the average inbreeding coefficient was acceptable, its increase in the subsequent years and the increasing percentage of inbred animals seems disturbing. The inbreeding coefficientlevels in modern sport horses should be monitored and pedigree information should be considered in breeding work.

## Data Availability Statement

The original contributions presented in the study are included in the article/Supplementary Material, further inquiries can be directed to the corresponding author/s.

## Ethics Statement

Ethical review and approval was not required for the animal study because Non-invasive population studies based on pedigree data.

## Author Contributions

TP: conceptualization and writing—original draft preparation. TP, SK, and GZ: methodology. GZ and IR-R: validation and supervision. TP and JB: formal analysis. TP and IR-R: investigation. KK: data curation. GZ, KK, and SK: writing—review and editing. TP and KD: visualization. TP and KK: project administration. All authors have read and agreed to the published version of the manuscript.

## Conflict of Interest

The authors declare that the research was conducted in the absence of any commercial or financial relationships that could be construed as a potential conflict of interest.

## Footnotes

1 https://data.fei.org/Calendar (Accessed December 3, 2019).
