# Supplementary material for: Pedigree Analysis of Warmblood Horses Participating in Competitions for Young Horses
Source: Front Genet. 2021 Apr 15;12:658403. doi: 10.3389/fgene.2021.658403 (PMC8082513; doi:10.3389/fgene.2021.658403)
Supplement: Supplementary file 1 [file Table_1.DOCX]

Table S1**.** Longest ancestral path (LAP).

| Ancestral path | Number of individuals | Ancestral path | Number of individuals |
| --- | --- | --- | --- |
| 0 | 1621 | 8 | 879 |
| 1 | 1233 | 9 | 718 |
| 2 | 1172 | 10 | 616 |
| 3 | 1147 | 11 | 499 |
| 4 | 1153 | 12 | 375 |
| 5 | 1088 | 13 | 239 |
| 6 | 1037 | 14 | 86 |
| 7 | 983 | 15 | 17 |
