# Supplementary material for: Pedigree Analysis of Warmblood Horses Participating in Competitions for Young Horses
Source: Front Genet. 2021 Apr 15;12:658403. doi: 10.3389/fgene.2021.658403 (PMC8082513; doi:10.3389/fgene.2021.658403)
Supplement: Supplementary file 2 [file Table_2.DOCX]

Table S2. Number of individuals, inbreds, unique ancestors, average inbreeding coefficients (%), average inbreeding coefficients in the inbreds (%), maximum and minimum inbreeding coefficients for the groups by birth year.

| Year  of birth | Number  of individuals | | | | Number  of inbreds | | | Number of unique ancestors | | | Average inbreeding coefficients (%) | | | Average inbreeding coefficients in the inbreds (%) | | | Max. of inbreeding coefficients (%) | | | Min. of inbreeding coefficients (%) | | |
| --- | --- | --- | --- | --- | --- | --- | --- | --- | --- | --- | --- | --- | --- | --- | --- | --- | --- | --- | --- | --- | --- | --- |
|  | Total | ♂♂ | ♀♀ | Total | | ♂♂ | ♀♀ | Total | ♂♂ | ♀♀ | Total | ♂♂ | ♀♀ | Total | ♂♂ | ♀♀ | Total | ♂♂ | ♀♀ | Total | ♂♂ | ♀♀ |
| 1999 | 17 | 10 | 7 | 13 | | 8 | 5 | 976 | 745 | 380 | 0.345 | 0.354 | 0.332 | 0.451 | 0.443 | 0.465 | 1.809 | 1.809 | 0.781 | 0.037 | 0.037 | 0.058 |
| 2000 | 44 | 30 | 14 | 25 | | 16 | 9 | 1719 | 1315 | 935 | 0.595 | 0.627 | 0.528 | 1.048 | 1.175 | 0.821 | 6.433 | 6.433 | 3.791 | 0.008 | 0.008 | 0.076 |
| 2001 | 94 | 73 | 21 | 53 | | 40 | 13 | 3041 | 2600 | 1210 | 0.543 | 0.532 | 0.579 | 0.962 | 0.971 | 0.936 | 7.010 | 7.010 | 3.134 | 0.001 | 0.001 | 0.043 |
| 2002 | 106 | 75 | 31 | 72 | | 51 | 21 | 3528 | 2961 | 1685 | 0.899 | 1.072 | 0.474 | 1.336 | 1.598 | 0.699 | 13.822 | 13.822 | 4.551 | 0.003 | 0.006 | 0.003 |
| 2003 | 110 | 79 | 31 | 84 | | 61 | 23 | 3911 | 3312 | 1828 | 0.921 | 1.087 | 0.492 | 1.217 | 1.426 | 0.664 | 13.455 | 13.455 | 4.330 | 0.001 | 0.006 | 0.001 |
| 2004 | 110 | 74 | 36 | 84 | | 58 | 26 | 3805 | 3060 | 1957 | 0.970 | 1.068 | 0.768 | 1.270 | 1.363 | 1.064 | 8.069 | 8.069 | 4.022 | 0.003 | 0.003 | 0.009 |
| 2005 | 87 | 63 | 24 | 75 | | 54 | 21 | 3322 | 2687 | 1689 | 1.260 | 1.295 | 1.168 | 1.462 | 1.511 | 1.335 | 7.440 | 6.583 | 7.440 | 0.006 | 0.006 | 0.021 |
| 2006 | 106 | 77 | 29 | 87 | | 62 | 25 | 3614 | 3129 | 1805 | 0.991 | 0.940 | 1.125 | 1.207 | 1.167 | 1.306 | 6.299 | 5.171 | 6.299 | 0.000 | 0.000 | 0.012 |
| 2007 | 110 | 73 | 37 | 97 | | 65 | 32 | 4238 | 3412 | 2209 | 1.121 | 1.097 | 1.169 | 1.271 | 1.232 | 1.352 | 8.070 | 6.667 | 8.070 | 0.003 | 0.004 | 0.003 |
| 2008 | 73 | 52 | 21 | 63 | | 44 | 19 | 3553 | 2982 | 1687 | 1.007 | 0.998 | 1.031 | 1.167 | 1.179 | 1.139 | 6.667 | 6.667 | 3.603 | 0.021 | 0.023 | 0.021 |
| 2009 | 79 | 51 | 28 | 73 | | 46 | 27 | 3598 | 2658 | 2129 | 1.410 | 1.657 | 0.960 | 1.526 | 1.837 | 0.996 | 9.193 | 9.193 | 2.914 | 0.000 | 0.000 | 0.004 |
| 2010 | 72 | 40 | 32 | 68 | | 39 | 29 | 3752 | 2666 | 2333 | 1.321 | 1.409 | 1.211 | 1.399 | 1.445 | 1.336 | 25.000 | 25.000 | 7.749 | 0.003 | 0.003 | 0.098 |
| 2011 | 40 | 27 | 13 | 36 | | 24 | 12 | 2703 | 2264 | 1287 | 1.270 | 1.314 | 1.179 | 1.411 | 1.478 | 1.276 | 5.423 | 0.054 | 0.041 | 0.001 | 0.000 | 0.000 |
| Total | 12863 | 4162 | 8701 | 3653 | | 1170 | 2483 | 0 | 0 | 0 | 0.465 | 0.465 | 0.465 | 1.638 | 1.638 | 0.016 | 31.348 | 0.313 | 0.313 | 0.000 | 0.000 | 0.000 |
