# Supplementary material for: Pedigree Analysis of Warmblood Horses Participating in Competitions for Young Horses
Source: Front Genet. 2021 Apr 15;12:658403. doi: 10.3389/fgene.2021.658403 (PMC8082513; doi:10.3389/fgene.2021.658403)
Supplement: Supplementary file 3 [file Table_3.docx]

Table S3. Most important founders in the analysed population.

| Founder  *Breed number* | Sex ^1^ | Breed ^2^ | Birth  year | cg  [%] | v_1_  [%] | m_1_  [%] | u_1_  [%] | v_2_  [%] | m_2_  [%] | u_2_[%] |
| --- | --- | --- | --- | --- | --- | --- | --- | --- | --- | --- |
| Ladykiller *DE306064000861* | S | xx | 1961 | 3.752 | 0.126 | 0.100 | 0.079 | 0.098 | 0.070 | 0.050 |
| Rantzau *DE306064779046* | S | xx | 1946 | 3.036 | 0.108 | 0.054 | 0.010 | 0.097 | 0.045 | 0.009 |
| Ramzes *DE321210365437* | S | xo | 1937 | 2.797 | 0.074 | 0.042 | 0.034 | 0.071 | 0.039 | 0.030 |
| Cottage Son *DE321210375544* | S | xx | 1944 | 2.591 | 0.071 | 0.045 | 0.039 | 0.061 | 0.033 | 0.027 |
| Furioso *DE306064703339* | S | xx | 1939 | 2.078 | 0.048 | 0.010 | 0.006 | 0.052 | 0.013 | 0.009 |
| Loretto *DE321210289432* | S | hol | 1932 | 1.986 | 0.045 | 0.025 | 0.023 | 0.040 | 0.020 | 0.018 |
| Vestale du Bois  *CE00040836* | M | sf | 1942 | 1.353 | 0.048 | 0.011 | 0.000 | 0.043 | 0.009 | 0.000 |
| Anblick *DE321210369138* | S | xx | 1938 | 0.897 | 0.015 | 0.005 | 0.002 | 0.014 | 0.004 | 0.002 |
| Fanatiker *DE321210321940* | S | hol | 1940 | 0.883 | 0.013 | 0.004 | 0.002 | 0.014 | 0.004 | 0.002 |
| Dame de Ranville *DE304046020147* | M | sf | 1947 | 0.873 | 0.009 | 0.003 | 0.001 | 0.010 | 0.004 | 0.002 |

^1^ S – sire, M – mare; ^2^ xx – thoroughbred, sf – selle francais, xo – half-bred Anglo-Arabian horse, hol – Holstein horse. Ancestor component vectors were calculated: cg - contribution of genes of founders; v_1_ - contribution of genes of founders to the average inbreeding coefficient; m_1_ - contributions of Mendelian sampling variances of ancestors to the average inbreeding coefficient; u_1_ - contributions of genes of nodal common ancestors to the average inbreeding coefficient; v_2_ - contribution of genes of founders to the average coancestry; m_2_ - contributions of Mendelian sampling variances of ancestors to the average coancestry; u_2_ - contributions of genes of nodal common ancestors to the average coancestry.
